# Synthesis, Structural Characterization, and Evaluation of the Biological Properties of Heteroleptic Palladium(II) Complexes

**DOI:** 10.1155/2014/916361

**Published:** 2014-09-07

**Authors:** Hizbullah Khan, Nek Daraz, Muhammad Nasim Khan, Muhammad Said, Nosheen Akhtar, Amin Badshah, Amir Sada Khan, Murad Ali

**Affiliations:** ^1^Department of Chemistry, University of Science and Technology, Bannu 28100, Pakistan; ^2^Department of Chemistry, Kohat University of Science and Technology, Kohat 26000, Pakistan; ^3^Department of Chemistry, Abdul Wali Khan University, Mardan 23200, Pakistan; ^4^Department of Chemistry, Quaid-i-Azam University, Islamabad 45320, Pakistan; ^5^PETRONAS Ionic Liquid Centre, Department of Chemical Engineering, Universiti Teknologi Petronas, Seri Iskandar, 31750 Tronoh, Perak, Malaysia

## Abstract

Five heteroleptic palladium(II) complexes of the general formula Pd(PR_3_)(tu)Cl_2_, where PR_3_ = triphenylphosphine (**1**), diphenyl-*o*-tolylphosphine (**2**), diphenyl-*p*-tolylphosphine (**3**), diphenyl-*t*-butylphosphine (**4**), and diphenyl-*o*-methoxyphenylphosphine (**5**), and tu = 1,3-bis(2-methoxyphenyl) thiourea. They all have been synthesized and characterized by various spectroscopic techniques (elemental analysis, FTIR, and ^1^H NMR and the ligand 1,3-bis(2-methoxyphenyl) thiourea was synthesized by single crystal X-ray diffraction technique). The synthesized compounds were screened for their antibacterial activity against four strains of bacteria (*Escherichia coli*, *Shigella flexneri*, *Staphylococcus aureus*, and *Bacillus subtilis*). The antitumor potential was evaluated in terms of activity against brine shrimp eggs and DNA interaction. The mixed ligand complexes have exhibited moderate antibacterial activity and promising antitumor potential.

## 1. Introduction

Metals medicinal applications are almost several hundred years old. But due to the lack of experience of pharmacologists and traditional medicinal chemists in dealing with biologically active metal complexes, the field of metal-based drugs could not flourish at the desired rate. However, it provides an opportunity to transition metal chemists to establish the development of exciting new drugs [1–5]. With the improvement in the understanding of metalloprotein function, some outstanding models of metal ion active sites and recent advances in discovering how naturally occurring metal ions are delivered to these active sites, and how metal ions are complexed in the reduction of some diseases; specify new roles for metal ions in curative strategies [6–10]. Although some nonmetal species like tamoxifen and taxol also show biological functions, it is clear that a major role is played by metal ions in biochemical process. Metal ions are considered to exert an inductive effect by coordination to the site of reaction and they work as reduction-oxidation sites that function either through electron or atom transfer [11–14]. The present development of successful metal based pharmaceuticals, which include the platinum anticancer drugs and radiodiagnostic agents, shows the usefulness of complexes as both diagnostic and therapeutic agents [15–19]. Cancer is one of the diseases that are responsible for the high death rate globally. Consequently, there has been a great need for continuous research to synthesize new drugs to control this disease.

During the mid-1970s, initial antitumor studies with* cis*-diamminedichloroplatinum(II) (cisplatin) indicated significant activity against some of the cancer cell-lines, especially leukemia L1210 tumors. Cisplatin remains to be the most effective drug in clinical treatment for ovarian, testicular, bladder, head, and neck cancers. In combination with other anticancer drugs such as doxorubicin, and 5-fluorouracil, it is widely used in treatment of neck cancer. However, there is a built-in and acquired resistance that limits the clinical usefulness of the drug. Platination of DNA is considered to be responsible for the antitumor activity of various platinum containing drugs through bonding to guanine [20, 21]. Palladium(II) analogues are structurally similar to those of Pt(II) complexes which have a very similar coordination geometry and coordination process [22–24]. Generally, Pt(II) complexes are less stable than Pd(II) compounds. Therefore, Pd(II) systems are frequently used as model complexes [25]. Pd(II) binding to the guanine ring has been proposed from IR and NMR data in several models. The models reveal coordination with C=O–Pd–N_7_ bridge between adjacent guanine rings and chelation through N_7_ and C=O of a guanine ring [26].

We report here the synthesis, spectral characterization, and biological activities of the heteroleptic Pd(II) complexes of the general formula Pd(PR_3_)(tu)Cl_2_, where PR_3_ = triphenylphosphine (**1**), diphenyl-*o*-tolylphosphine (**2**), diphenyl-*p*-tolylphosphine (**3**), diphenyl-*t*-butylphosphine (**4**), and diphenyl-*o*-methoxyphenylphosphine (**5**); tu = 1,3-bis(2-methoxyphenyl) thiourea.

## 2. Experimental

### 2.1. Materials and Methods

Ethanol, methanol,* n*-hexane, fish sperm DNA, dichloromethane, chloroform, carbon disulfide and PdCl_2_, sea salt, and brine shrimp eggs were obtained from Merck Germany and Sigma-Aldrich. All the solvents and chemicals were of analytical grade and were used as received, without further purification.

### 2.2. Synthesis of the Substituted Thiourea

**Scheme 1:**
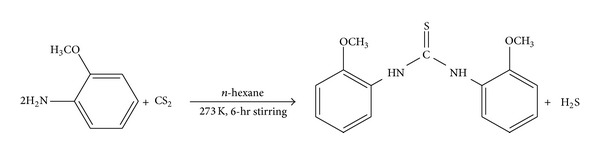
Synthesis of the substituted thiourea.

Substituted thiourea was synthesized by the reaction of* o*-methoxyphenylamine with carbon disulphide (2 : 1), respectively, at 273 Kelvin in* n*-hexane, as shown in Scheme 1, by the reported literature method with slight modification [27]. The reaction mixture was magnetically stirred for six hours and the reaction progress was continuously monitored by thin layer chromatography. The solvent was evaporated under the reduced pressure and the light brown solid was dissolved in a mixture of dichloromethane and* n*-hexane (2 : 1 v/v) for recrystallization. Needle shaped light brown crystals were obtained by the slow evaporation of the solvents at room temperature, and a suitable crystal was separated for single crystal diffraction analysis.

### 2.3. Synthesis of the Complexes** (**1–5**)**

The mixed ligand Pd(II) complexes were synthesized in two steps [28]. In the first step, Pd-organophosphine complex was formed by reacting organophosphine ligand, dissolved in acetone, with palladium chloride dissolved in dry methanol and few drops of concentrated hydrochloric acid in 2 : 1 molar ratio. The solid golden yellow product was obtained by evaporating the reaction mixture at reduced pressure. In the second step, the golden yellow product was dissolved in dichloromethane and was reacted with 1,3-bis(2-methoxyphenyl) thiourea solution in dichloromethane in 1 : 1 molar ratio. The final heteroleptic Pd(II) complex was obtained by rotary evaporation of the reaction mixture.

#### 2.3.1. [Pd(1,3-bis(2-methoxyphenyl) thiourea)(PPh_3_)Cl_2_]** (**1**)**

Quantities used were 0.32 g (0.47 mmol) of Pd(PPh_3_)_2_Cl_2_ and 0.15 g (0.47 mmol) of 1,3-bis(2-methoxyphenyl) thiourea in 25 mL of dichloromethane. Yield: 0.29 g (87%). M.p.: 231-232°C. FTIR (powder, cm^−1^): 3316 *υ*(N–H), 1122 *υ*(C–N), 622 *υ*(C=S), 2831 *υ*(C–H, aliphatic), and 2943 *υ*(C–H, aromatic). ^1^H NMR (300 MHz, CDCl_3_) *δ*-ppm: 3.72 (s, 3H, –OCH_3_), 4.05 (s, 2H, –NH), and 6.35–7.59 (m, 23H, Ar–H). Anal. Calc. for C_33_H_31_Cl_2_N_2_O_2_PPdS: C, 54.45; H, 4.29; N, 3.85; S, 4.40. Found: C, 54.50; H, 4.26; N, 3.87; S, 4.40.

#### 2.3.2. [Pd(1,3-bis(2-methoxyphenyl) thiourea)(PPh_2_-*o*-tolyl)Cl_2_]** (**2**)**

Quantities used were 0.35 g (0.47 mmol) of Pd(PPh_2_-*o*-tolyl)_2_Cl_2_ and 0.15 g (0.47 mmol) of 1,3-bis(2-methoxyphenyl) thiourea in 25 mL of dichloromethane. Yield: 0.30 g (85%). M.p.: 237-238°C. FTIR (powder, cm^−1^): 3313 *υ*(N–H), 1022 *υ*(C–N), 633 *υ*(C=S), 2832 *υ*(C–H, aliphatic), and 2942 *υ*(C–H, aromatic). ^1^H NMR (300 MHz, CDCl_3_) *δ*-ppm: 2.33 (s, 3H, –CH_3_), 3.75 (s, 3H, –OCH_3_), 4.00 (s, 2H, –NH), and 6.33–7.63 (m, 22H, Ar–H). Anal. Calc. for C_34_H_33_Cl_2_N_2_O_2_PPdS: C, 55.03; H, 4.48; N, 3.78; S, 4.32. Found: C, 55.10; H, 4.47; N, 3.78; S, 4.33.

#### 2.3.3. [Pd(1,3-bis(2-methoxyphenyl) thiourea)(PPh_2_-*p*-tolyl)Cl_2_]** (**3**)**

Quantities used were 0.34 g (0.47 mmol) of Pd(PPh_2_-*p*-tolyl)_2_Cl_2_ and 0.15 g (0.47 mmol) of 1,3-bis(2-methoxyphenyl) thiourea in 25 mL of dichloromethane. Yield: 0.32 g (91%). M.p.: 248-249°C. FTIR (powder, cm^−1^): 3318 *υ*(N–H), 1021 *υ*(C–N), 640 *υ*(C=S), 2832 *υ*(C–H, aliphatic), and 2944 *υ*(C–H, aromatic). ^1^H NMR (300 MHz, CDCl_3_) *δ*-ppm: 2.34 (s, 3H, –CH_3_), 3.70 (s, 3H, –OCH_3_), 4.02 (s, 2H, –NH), and 6.30–7.65 (m, 22H, Ar–H). Anal. Calc. for C_34_H_33_Cl_2_N_2_O_2_PPdS: C, 55.03; H, 4.48; N, 3.78; S, 4.32. Found: C, 55.09; H, 4.44; N, 3.77; S, 4.29.

#### 2.3.4. [Pd(1,3-bis(2-methoxyphenyl) thiourea)(PPh_2_-*t*-butyl)Cl_2_]** (**4**)**

Quantities used were 0.31 g (0.47 mmol) of Pd(PPh_2_-*t*-butyl)_2_Cl_2_ and 0.15 g (0.47 mmol) of 1,3-bis(2-methoxyphenyl) thiourea in 25 mL of dichloromethane. Yield: 0.27 g (81%). M.p.: 269-270°C. FTIR (powder, cm^−1^): 3321 *υ*(N–H), 1023 *υ*(C–N), 647 *υ*(C=S), 2831 (C–H, aliphatic), and 2945 *υ*(C–H, aromatic). ^1^H NMR (300 MHz, CDCl_3_) *δ*-ppm: 2.42 (s, 9H, –C(CH_3_)_3_), 3.69 (s, 3H, –OCH_3_), 4.01 (s, 2H, –NH), and 6.37–7.35 (m, 18H, Ar–H). Anal. Calc. for C_31_H_35_Cl_2_N_2_O_2_PPdS: C, 52.58; H, 4.98; N, 3.96; S, 4.52. Found: C, 52.49; H, 4.97; N, 3.96; S, 4.51.

#### 2.3.5. [Pd(1,3-bis(2-methoxyphenyl) thiourea)(PPh_2_-*o*-methoxyphenyl)Cl_2_]** (**5**)**

Quantities used were 0.36 g (0.47 mmol) of Pd(PPh_2_-*t*-butyl)_2_Cl_2_ and 0.15 g (0.47 mmol) of 1,3-bis(2-methoxyphenyl) thiourea in 25 mL of dichloromethane. Yield: 0.30 g (84%). M.p.: 252-253°C. FTIR (powder, cm^−1^): 3328 *υ*(N–H), 1022 *υ*(C–N), 636 *υ*(C=S), 2835 *υ*(C–H, aliphatic), and 2946 *υ*(C–H, aromatic). ^1^H NMR (300 MHz, CDCl_3_) *δ*-ppm: 3.70 (s, 9H, –OCH_3_) and 4.01 (s, 2H, –NH). Anal. Calc. for C_31_H_35_Cl_2_N_2_O_2_PPdS: C, 53.87; H, 4.39; N, 3.70; S, 4.23. Found: C, 53.75; H, 4.38; N, 3.69; S, 4.20.

### 2.4. Structural Study of the Ligand 1,3-Bis(2-methoxyphenyl) Thiourea

The crystal structure of the thiourea derivative was developed by the evaporation method, from the solvent of methanol. Colorless needle shaped crystals were obtained after slow evaporation of the solvent from a methanolic solution of the ligand at room temperature. The needle shaped crystal was mounted on a glass fiber using epoxy glue. The measurements were made at 293 K on a Bruker APEX II area detector diffractometer equipped with graphite monochromatic Mo-Kα radiation (0.71073 Å). The programme used for retrieving the cell parameters and data collection was APEX [29]. The data were integrated using the programme SAINT [30] and were corrected for Lorentz and polarization effects. The structure was solved and refined using SHELXS-97 and SHELXL-97 [31]. All the non-H atoms were refined anisotropically. The hydrogen atoms were placed at the idealized positions. The crystal data and structure refinement parameters are shown in Table 1.

### 2.5. Biological Assays

#### 2.5.1. Antibacterial Assay

All the synthesized metal complexes were investigated for their antibacterial activity against four strains of bacteria (*Escherichia coli*,* Shigella flexneri*,* Staphylococcus aureus*, and* Bacillus subtilis*). The agar well diffusion method [32] was used to determine the inhibition zone and minimum inhibitory concentration (MIC). Broth culture (0.70 mL) containing* ca.*, 10^6^ colony forming units (CFU) per mL of the test strain was added to 70 mL of nutrient agar medium at 45°C, mixed well, and then poured into a 14 cm diameter sterile petri plate. The media was allowed to solidify and 8 mm wells were dug by a sterile metallic borer. DMSO test sample (100 *μ*L) at 1 mg/mL was added to the respective wells. DMSO was acting as a negative control, while the standard drug Imipenem (10 *μ*g/disc) was used as a positive control. Triplicate plates of each bacterial strain were prepared and incubated aerobically at 37°C for 24 hours. The activity was determined by measuring the diameter of zone (mm) with the aid of a Vernier Caliper (precision ± 0.1 mm). The growth inhibition was calculated with reference to the positive control.

#### 2.5.2. Cytotoxicity Screenings

Stock solutions of the compounds were prepared by dissolving 5 mg/5 mL in methanol. From the stock solution three other solutions of 50 *μ*g/mL, 100 *μ*g/mL, and 500 *μ*g/mL concentrations were prepared by using the dilution formula. As the assay was carried out in duplicate, each solution was divided into two parts thus preparing three other solutions, 50 *μ*g/mL, 100 *μ*g/mL, and 500 *μ*g/mL, respectively. Two test tubes for control were also prepared having only 5 mL of media.

An amount of 2.8 g of sea salt was dissolved in 100 mL of distilled water (2.8 g/100 mL) and put on the magnetic stirrer for two hours. A rectangular tray having many pores in its partition was taken for hatching the shrimps. The media and shrimp eggs were put in the tray covered with aluminum foil provided with an air pump. After 24 hours incubation (using table lamp), the larvae were shifted from the darkened side to the open portion of the tray. The larvae were collected by poster pipette.

A volume of 0.5 mL (500 *μ*L) of each solution was taken in a separate test tube labeled as 50 *μ*g/mL, 100 *μ*g/mL, and 500 *μ*g/mL, and eight larvae were transferred to each test tube with the help of a poster pipette dropper. The solution was made 5 mL by the addition of media. A separate test tube containing only 5 mL media was also taken and labelled as control. After taking all the necessary measures, each test tube was tightly packed with cotton and incubated at 28°C for 24 hours. After 24 hours incubation, the number of living shrimps was counted and calculations were made by using Abbot's formula and the results are shown in Table 3

(1) $$\begin{array}{c} \%\;\text{death}=\left(\text{control}-\frac{\text{sample}}{\text{control}}\right)\times 100. \end{array}$$

### 2.6. Absorption Spectroscopy

At room temperature, absorption spectra were recorded on Shimadzu UV-1800 Koyoto Japan. Standard quartz cells of 1-cm path length were used. The concentration of the complex solution was kept constant at 2 *μ*M, while the concentration of the DNA solution was changed, by 20 *μ*L after each addition, in such a way that the total volume of the blank and the complex was constant. The change in the absorption spectra of the complexes with the increasing concentration of the DNA is shown in Table 4.

## 3. Results and Discussions

### 3.1. Structural Study of the Ligand 1,3-Bis(2-methoxyphenyl) Thiourea

**Figure 1:**
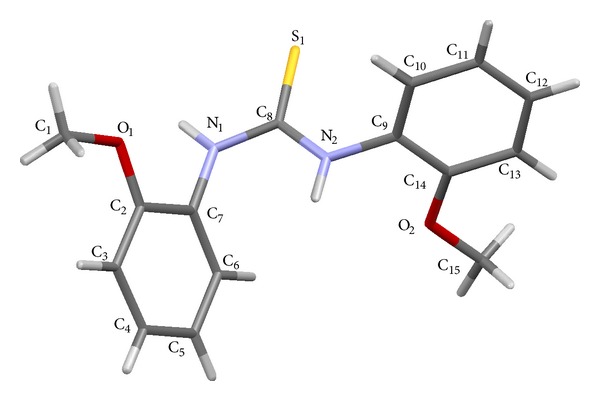
The capped sticks diagram of 1,3-bis(2-methoxyphenyl) thiourea. Selected bond lengths (Å) and angles (deg.): S_1_–C_8_ (1.690(2)), N_1_–C_8_ (1.352(2)), N_2_–C_8_ (1.342(3)), N_1_–C_7_ (1.429(2)), N_2_–C_9_ (1.432(3)), O_1_–C_1_ (1.426(3)), O_1_–C_2_ (1.367(3)), O_2_–C_14_ (1.370(3)), O_2_–C_15_ (1.429(3)), C_15_–O_2_–C_14_ (118.0(2)), C_1_–O_1_–C_2_ (118.3(2)), S_1_–C_8_–N_1_ (120.2(1)), S_1_–C_8_–N_2_ (122.9(1)), and N_1_–C_8_–N_2_ (116.9(2)).

The capped sticks diagram of the substituted thiourea with selected bond lengths and angles is shown in Figure 1. The carbon atom in N–C=S moiety of the ligand is sp^2^ hybridized. The compound crystallizes in C_2_/c space group. The C_8_–S_1_ bond length, 1.690(2) Å is greater than C=S double bond (1.60 Å), but shorter than C–S single bond (1.82 Å) distance [33, 34]. The torsion angle measurements show that C_1_O_1_C_2_C_3_ are almost planar with the torsion angle of −2.3(3)°. Similarly, the other methoxy group is lying in more planarity with the aromatic ring with the torsion angle of −1.1(3)° (C_15_O_2_C_14_C_13_).

### 3.2. Synthesis of the Complexes and Their Characterization

**Scheme 2:**
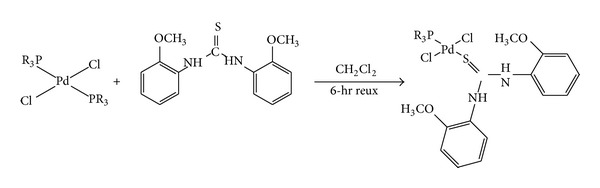
Synthesis of heteroleptic Pd(II) complexes.

Mixed ligand Pd(II) complexes were synthesized by reacting substituted thiourea ligand with the palladium phosphine complexes as shown in Scheme 2.

### 3.3. Infrared Spectroscopy

According to the literature, tentative assignments were made [35] and the appearance of the signal in the range of 3313–3328 cm^−1^ demonstrates the presence of N–H group in the complexes. The IR peak in the range of 1021–1122 cm^−1^ confirms the C–N bond and the 622–647 cm^−1^, shift shows the presence of C=S peak and aliphatic and aromatic peaks were observed in the respective regions. After the complexation, the C=S peak was slightly shifted to a lower frequency value which indicates the bond formation between the C=S group and the palladium moiety.

### 3.4. ^1^H NMR Study

The proton NMR spectra of the complexes confirm all the protons present in the complexes. The N–H peak was observed in the range of 4.00–4.05 ppm and a singlet peak in the range of 3.69–3.75 shows the OCH_3_ group and the aromatic protons were observed as multiplets in the range of 6.33–7.63, in the synthesized metallopharmaceutical drugs. The presence of all the protons was confirmed by the proton NMR, which ensures the complex formation between the ligand and the metal atom.

### 3.5. Cytotoxicity

The results show that concentration of the complexes is directly proportional to % death of brine shrimps. The maximum % death is shown by 500 *μ*g/mL; the 100 *μ*g/mL concentration showed moderate cytotoxicity and the sample 50 *μ*g/mL showed minimum cytotoxic activity. The compound** C**_**5**_ (Pd(1,3-bis(2-methoxyphenyl) thiourea)(PPh_2_-*o*-methoxyphenyl)Cl_2_) is the least cytotoxic while the complex** C**_**2**_ (Pd(1,3-bis(2-methoxyphenyl) thiourea)(PPh_2_-*o*-tolyl)Cl_2_) has exhibited maximum cytotoxicity. The least cytotoxicity of the complex** C**_**5**_ may be assigned to the active methoxy group present in the organophosphine group, which is sufficiently reactive to react with other groups before reaching the target site.

### 3.6. DNA Interaction

Electronic absorption spectroscopyisone of the versatile techniques to evaluate the interaction mode of complex and DNA molecule. In general, the hypochromism and the red shift reveal the intercalation mode of interaction involving a strong stacking interaction between the aromatic chromophore of the complex and the base pairs of DNA [36]. The data in Table 3 show that there might be some interactions between the drug molecule and DNA, revealed by the brine shrimp activity. Hyperchromism has been observed, which exhibits that the DNA interaction is not the classical intercalation mode, rather it is a groove binding [37]. The groove binding is generally shown by the molecules having unfused aromatic rings linked by bonds with torsional freedom that molecule may adopt conformations which are closely matched with the helical turn of DNA groove [38]. As far as the mixed-ligand Pd(II) complexes are concerned, it is clear that these compounds have unfused rings in the organophosphine ligands that may provide the Vander Waal's interaction in the helical DNA grooves.

### 3.7. Antibacterial Activity

The synthesized metallodrugs were screened for their antibacterial activity against two gram negative (*E. coli* and* S. flexneri*) and two gram positive (*B. subtilis* and* S. aureus*) bacterial strains, the results are presented in Table 2. All the metal complexes are less active than the standard drug Imipenem, however, they have shown moderate antimicrobial activity. The compound** 5** (Pd(1,3-bis(2-methoxyphenyl) thiourea)(PPh_2_-*o*-methoxyphenyl)Cl_2_) has shown maximum activity while compound** 3** (Pd(1,3-bis(2-methoxyphenyl) thiourea)(PPh_2_-*p*-tolyl)Cl_2_) is the least active compound of all the five synthesized compounds. The antibacterial results indicate that variation in the phosphine ligand changes the activity considerably. Generally, the compounds having the* tertiary*-butyl,* ortho*-tolyl and* para*-tolyl groups have less activity as compared to the compounds having triphenylphosphine and diphenyl-*o*-methoxyphenyl groups.

## 4. Conclusions

Five heteroleptic Pd(II) complexes have been synthesized and characterized by elemental analysis, FT-IR, and ^1^H NMR and the ligand thiourea derivative has also been characterized by single crystal X-ray diffraction. The synthesized compounds have exhibited moderate antibacterial activity against the four strains of bacteria. The cytotoxicity screening and the DNA binding interaction reveal that the compounds have promising antitumor potential.

## Figures and Tables

**Table 1:** Crystal data and details of the structure refinement of the ligand 1,3-bis(2-methoxyphenyl) thiourea.

Molecular formula	C_15_H_16_N_2_O_2_S
Molecular weight	288.36
Space group	C_2_/c
Cell lengths (Å)	**a**: 14.442
	**b**: 13.032
	**c**: 16.187
Cell angles (°)	**α**: 90.00
	***β***: 103.89
	***γ***: 90.00
Cell volume (Å)^3^	2957.44
Z, Z′	**Z**: 8
	**Z**′: 0
*R*-factor (%)	2.91
Temperature	293(2) K
Wavelength	0.71073 Å

**Table 2:** Antibacterial activity of the complexes* (in vitro)* against four different bacterial pathogens (diameter of inhibition zone in mm).

Sample codes	*E. coli *	*B. subtilis *	*S. flexneri *	*S. aureus *
**1**	18	19	20	23
**2**	19	17	15	22
**3**	14	14	18	18
**4**	18	13	16	24
**5**	20	17	19	25
Standard Drug, Imipenem	30	32	35	40

Conc. of the standard drug “Imipenem” = 10 *μ*g/disc.

Concentration of sample = 5 mg/mL (stock solution) and 10 *μ*g/disc.

**Table 3:** The number of surviving shrimps after 24 hours incubation in the complexes and control.

Compounds	50 *μ*g/mL	100 *μ*g/mL	500 *μ*g/mL	Control	Mean % death
**1**	6	5	2	8	45.83 ± 21.24
**2**	3	1	0	8	83.33 ± 15.60
**3**	5	3	2	8	45.83 ± 25.12
**4**	4	3	2	8	70.83 ± 13.17
**5**	7	4	3	8	41.67 ± 21.24

**Table 4:** UV data of Pd(II) complexes of the type formula [Pd(PR_3_)(tu)Cl_2_].

S. number	Code of the complex	*λ*_max⁡_ of the complex	*λ*_max⁡_ of the complex after DNA addition	*μ*L of DNA added and absorbance at *λ*_max⁡_			
				20 *μ*L	40 *μ*L	60 *μ*L	80 *μ*L
1	**C**_1_	306	304	1.132	1.138	1.145	1.151
2	**C**_2_	332	329	0.964	0.975	0.979	0.987
3	**C**_3_	290	285	1.441	1.459	1.463	1.470
4	**C**_4_	304	303	1.422	1.459	1.463	1.470
5	**C**_5_	308	304	0.943	0.947	0.952	0.957

## Additional Data

The crystallographic data for the structural analyses of the ligand 1,3-bis(2-methoxyphenyl) thiourea has been deposited with the Cambridge Crystallographic Data Centre, CCDC no. CCDC 929755. Copy of this information may be obtained free of charge from the Director, CCDC, 12 Union Road, Cambridge, CBZ 1EZ, UK (fax: +44 1223 336 033; email: deposit@ccdc.cam.ac.uk or http://www.ccdc.cam.ac.uk).
